# C-951-08. Randomized Control Trial Comparing Methods of Pain Control for Skin Graft Donor Sites

**DOI:** 10.1093/jbcr/irag033.116

**Published:** 2026-04-06

**Authors:** Ellen L Nangia, Cole L Bird, Divya Hegde, Julia C Slater, Duncan Nickerson, Jessica Reynolds, Dhaval Bhavsar

**Affiliations:** The University of Kansas Health System Burnett Burn Center, Kansas City, KS; The University of Kansas Health System Burnett Burn Center, Kansas City, KS; The University of Kansas Health System Burnett Burn Center, Kansas City, KS; The University of Kansas Health System Burnett Burn Center, Kansas City, KS; The University of Kansas Health System Burnett Burn Center, Kansas City, KS; The University of Kansas Medical Center, Kansas City, KS; The University of Kansas Medical Center, Kansas City, KS

## Abstract

**Introduction:**

Over 160 000 skin grafts are performed annually in The United States. Opioids remain the mainstay treatment of postsurgical pain despite their numerous adverse effects. Alternative analgesic strategies in lieu of the current standard of care (SOC) are proposed to improve pain control in graft donor sites and reduce opioid burden.

**Methods:**

A randomized control trial was conducted with patients 18 years of age and older admitted to an accredited burn center. Patients with burn injuries ≤5% total body surface area (TBSA) of deep partial/full thickness involvement were undergoing their first autografting procedure with donor sites limited to the anterior or lateral thigh. Phase 1 of the trial included 50 patients with a 1:1 blinded randomization to local liposomal bupivacaine infiltration or SOC local lidocaine infiltration. Phase 2 enrolled an additional 24 patients assigned to receive a regional nerve block, totaling 74 subjects. Postoperative pain was assessed using the Visual Analog Scale (VAS), and opioid use was quantified in morphine milligram equivalents (MME); both measures were recorded starting 24 hours preoperative to postoperative day three.

**Results:**

Patients who received a regional nerve block in Phase 2 required a lower initial postoperative opioid dose compared to those receiving lidocaine (*p*=.0018). First pain scores recorded postoperatively were lower in the nerve block cohort (*p*=.0051) as well as scores taken on day three (*p*=.0236) (Table 1). No differences were found in regard to MME within Phase 2. Phase 1 comparing bupivacaine to lidocaine infiltration identified no significant differences.

**Conclusions:**

The use of nerve block demonstrated a trend in reducing pain postoperatively compared to current SOC lidocaine infiltration. On average, pain scores in the nerve block cohort were lower than lidocaine, displaying clinical relevance despite absence of statistical significance which may reflect the limited sample size.

**Applicability of Research to Practice:**

As regional nerve blocks are an effective strategy to manage postoperative pain in skin graft donor sites and reduce reliance on opioids, they should be more commonly utilized in clinical practice. Reduction of opioid requirements in the inpatient setting, and therefore decreasing opioid burden and adverse effects, will enhance patient recovery and exposure amid the broader efforts to address the ongoing opioid crisis.

**Funding for the study:**

N/A.

